# VRK2 activates TNFα/NF-κB signaling by phosphorylating IKKβ in pancreatic cancer

**DOI:** 10.7150/ijbs.66313

**Published:** 2022-01-09

**Authors:** Jionghuang Chen, Kexiong Qiao, Chaolei Zhang, Xinyang Zhou, Qian Du, Yuezhen Deng, Liping Cao

**Affiliations:** 1 Department of General Surgery, Sir Run Run Shaw Hospital, Zhejiang University School of Medicine, Hangzhou, China.; 2 Xiangya Cancer Center, Xiangya Hospital, Central South University, Changsha, China.

**Keywords:** Pancreatic cancer, VRK2, NF-κB signaling, Phosphorylation, Organoid

## Abstract

NF-κB signaling is active in more than 50% of patients with pancreatic cancer and plays an important role in promoting the progression of pancreatic cancer. Revealing the activation mechanism of NF-κB signaling is important for the treatment of pancreatic cancer. In this study, the regulation of TNFα/NF-κB signaling by VRK2 (vaccinia-related kinase 2) was investigated. The levels of VRK2 protein were examined by immunohistochemistry (IHC). The functions of VRK2 in the progression of pancreatic cancer were examined using CCK8 assay, anchorage-independent assay, EdU assay and tumorigenesis assay. The regulation of VRK2 on the NF-κB signaling was investigated by immunoprecipitation and invitro kinase assay. It was discovered in this study that the expression of VRK2 was upregulated in pancreatic cancer and that the VRK2 expression level was significantly correlated with the pathological characteristics and the survival time of patients. VRK2 promoted the growth, sphere formation and subcutaneous tumorigenesis of pancreatic carcinoma cells as well as the organoid growth derived from the pancreatic cancer mouse model. Investigation of the molecular mechanism indicated that VRK2 interacts with IKKβ, phosphorylating its Ser177 and Ser181 residues and thus activating the TNFα/NF-κB signaling pathway. An IKKβ inhibitors abolished the promotive effect of VRK2 on the growth of organoids. The findings of this study indicate that VRK2 promotes the progression of pancreatic cancer by activating the TNFα/NF-κB signaling pathway, suggesting that VRK2 is a potential therapeutic target for pancreatic cancer.

## Introduction

TNFα is highly expressed in 50% of patients with pancreatic ductal adenocarcinoma (PDAC) 1, 2. This observation indicates that the canonical NF-κB signaling pathway is active in pancreatic cancer 3. Inhibiting the transcriptional activity of NF-κB inhibits angiogenesis and metastasis in pancreatic cancer 4, 5. Multiple studies indicate that targeting the canonical NF-κB signaling pathway significantly improves the treatment of pancreatic cancer 6, 7. The NF-κB signaling pathway activated by TNFα plays a rather important role in the initiation and progression of pancreatic cancer 8. The key event in TNFα-mediated NF-κB signaling is the activation of the IKK complex 9. The IKK complex comprises the IKKα, IKKβ and IKKγ. IKKα and IKKβ are the catalytic subunits of this complex, while IKKγ is the regulatory subunit 9. Gene disruption experiments indicate that IKK activity and classical NF-kB activation are completely dependent on the integrity of IKKγ 10. Of the two catalytic subunits, the most important for activation of the classical NF-kB signaling pathway is IKK-β^11^-^13^. Interestingly, cells lacking IKKα show normal induction of NF-κB DNA-binding activity in response to most stimuli 14, 15. Activation of IKK complex depends on its phosphorylation at Ser177 and Ser181 (Ser176 and Ser180 in IKKα) in the activation loop of IKKβ 16. In the inactivated state, NF-κB is bound by IκB and is retained in the cytoplasm 16. TNFα treatment activates the kinase subunits in the IKK complex, and the IKK complex phosphorylates the IκB protein 17. Phosphorylation of IκB leads to its ubiquitination and proteasome degradation, leading to the release of NF-κB/Rel complex 17. The active NF-κB/Rel complex is further activated by posttranslational modifications (phosphorylation, acetylation, and glycosylation) and transported into the nucleus, where it functions alone or with other transcription factors, including AP-1, Ets, and Stat, to induce target gene expression 18. Activation of NF-κB signaling helps to promote acinar-to-ductal metaplasia (ADM), forming the PanIN (Pancreatic intraepithelial neoplasia) 19. In pancreatic cancer cells, activation of NF-κB signaling promotes cell growth and inhibits pancreatic cell apoptosis 20, 21. In 90% of PDAC cases, the Kras is constitutively activated 22. Previous studies indicate that in Kras^G12D^-driven pancreatic cancer, inactivation of IKKβ inhibits the malignant progression 23. This observation suggests that targeting IKKβ is of great importance for the treatment of pancreatic cancer. However, the existing IKKβ inhibitors (e.g., ML-120B and TPCA1) cannot be used in clinical practice owing to the toxic side effects observed in the preclinical model 24, 25. Therefore, for the development of IKKβ inhibitors, it is of great importance to reveal the regulatory mechanism of IKKβ in pancreatic cancer. It is generally believed that TAK1 is responsible for phosphorylating IKKα and IKKβ 26. It remains unclear whether there is any unidentified kinase in pancreatic cancer that phosphorylates IKKα and IKKβ.

There are three vaccinia-related kinases (VRKs): VRK1, VRK2, and VRK3 27, 28. VRK1 and VRK3 are localized in the nucleus, while VRK2 is localized in the cytoplasm 28. VRK2 encodes a serine-threonine protein kinase that shares 38.7% amino acid sequence homology with the B1R serine-threonine protein kinase 28. The studies on the biological functions of VRK2 have revealed that it regulates apoptosis in a variety of ways. VRK2 can phosphorylate Thr18 of P53 to promote the stability of P53 and inhibit its ubiquitination by MDM2 29. On the other hand, VRK2 interacts with Bcl-xl to inhibit apoptosis 30. VRK2 interacts with BHRF1, an Epstein-Barr virus (EBV) early gene product, in EBV-positive nasopharyngeal carcinoma cells 31. In addition, VRK2 can phosphorylate multiple transcription factors to change the transcription of genes. VRK2 phosphorylates Ser32 of NFAT1, promoting COX2 transcription and tumor cell infiltration 32. VRK1 and VRK2 phosphorylate the Ser/Thr2-4 residues of Barrier to Autointegration Factor (BAF), thereby abolishing the ability of the BAF protein to bind to DNA, making the BAF protein unable to be retained in the nucleus 33. VRK2 can also function independently of its kinase activity. In a hypoxic environment, VRK2 can inhibit TAK1-JNK signaling cascades without relying on its kinase activity 34. VRK2 can anchor KSR1 and MEK1 into the endoplasmic reticulum to achieve spatial regulation of this MAPK kinase 35. In addition to its role in tumor progression, VRK2 kinase activity is inhibited by GSK3β in Huntington's disease, thus reducing polyglutamine aggregation 36. Currently, the role of VRK2 in pancreatic cancer is poorly understood.

In this study, the expression pattern of VRK2 in pancreatic cancer was studied. Additionally, both the roles of VRK2 in the progression of pancreatic cancer and the related mechanism were investigated.

## Results

### The expression of VRK2 is upregulated in pancreatic cancer, and the VRK2 expression level is significantly correlated with the pathological characteristics of pancreatic cancer

**Figure 1:**
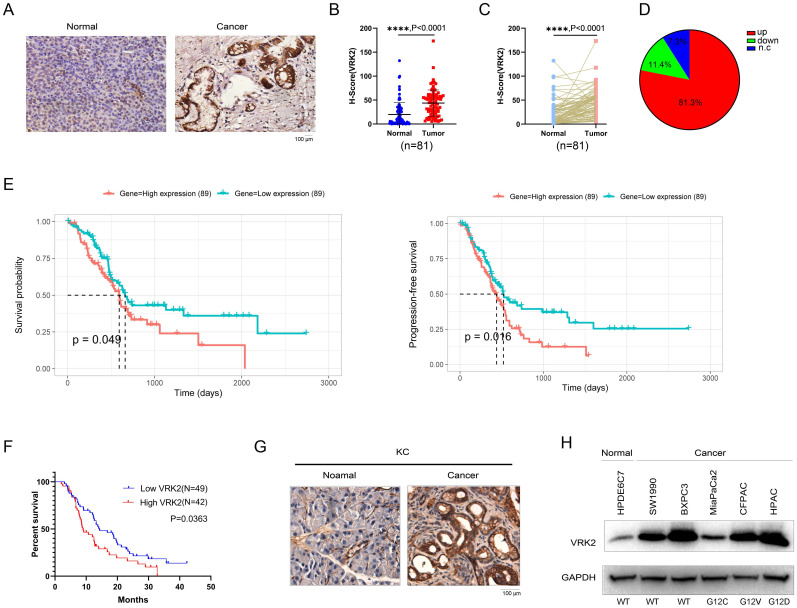
** VRK2 is upregulated in pancreatic cancer.** (**A**) Representative images of IHC staining to examine the protein level of VRK2 in pancreatic cancer tissues (Cancer) and adjacent noncancerous tissues (Normal). (**B**) The H-score of VRK2 IHC staining. The tissue array was scored using the Vectra 2 system. The H-scores of VRK2 in cancer tissues and adjacent noncancerous tissues were statistically analyzed. (**C**) The H-score of VRK2 IHC staining. The tissue array was scored as described in the “Materials and methods”. The H-scores of VRK2 in cancer tissues and adjacent noncancerous tissues from the same patient were statistically analyzed. (**D**) The pie chart shows the percentage of patients with the upregulation or downregulation of VRK2 in their cancer tissues. N.c, no change. (**E**) The survival curve derived from the TCGA database. (**F**) Kaplan-Meier survival analysis was performed to evaluate the correlation between the expression of VRK2 and survival in the tissue array. (**G**) IHC was performed to examine the expression of VRK2 in the KC mouse model (Pdx-Cre; Kras^G12D^). (**H**) Western blotting was performed to examine the VRK2 protein level in pancreatic cancer cell lines and a normal ductal epithelial cell line (HPDE6C7). ****, *P* < 0.0001.

To reveal the expression pattern of VRK2 in pancreatic cancer, the protein levels of VRK2 in the tissue array (containing 81 pancreatic cancer tissues and paired adjacent tissues) were first measured using immunohistochemistry (IHC). The IHC results showed that compared with that in the adjacent tissues, the protein level of VRK2 was increased in the pancreatic cancer tissues (Fig. 1A). By analyzing the IHC score of VRK2 in the adjacent tissues and pancreatic cancer tissues, it was found that the protein level of VRK2 was higher in the pancreatic cancer tissues (Fig. 1B). In addition, the protein levels of VRK2 in the tumor tissues and adjacent tissues from the same patient were compared (Fig. 1C), and the results showed that in 81.3% of the patients, there was a higher level of VRK2 protein in the pancreatic cancer tissues (Fig. 1D). Moreover, the protein level of VRK2 in the pancreatic cancer tissues was significantly correlated with pathological parameters, such as tumor size and smoking status (Table 1). Furthermore, the correlation between the expression of VRK2 and survival time was investigated. In the TCGA database, higher VRK2 expression was found to associate with a shorter survival time (Fig. 1E), and similar results were found in the tissue array (Fig. 1F). KC (LSL-Kras^G12D^; Pdx-Cre) mice are one of the widely used pancreatic cancer models. The expression of Vrk2 in pancreatic tissues was evaluated, and the results showed that there was a higher level of Vrk2 protein in the tumor tissues (Fig. 1G). Finally, the expression of VRK2 in HPDE6C7 (normal pancreatic cells) and a panel of pancreatic cancer cells was evaluated. The results showed that VRK2 was expressed at low levels in HPDE6C7 cells and at high levels in pancreatic cancer cells (Fig. 1H). Taken together, these observations suggest that the expression of VRK2 is upregulated in pancreatic cancer and that VRK2 is therefore very likely to play an important role in pancreatic cancer.

### VRK2 promotes the growth, colony formation, and sphere formation of pancreatic cancer cells and enhances their tumorigenicity in nude mice

**Figure 2:**
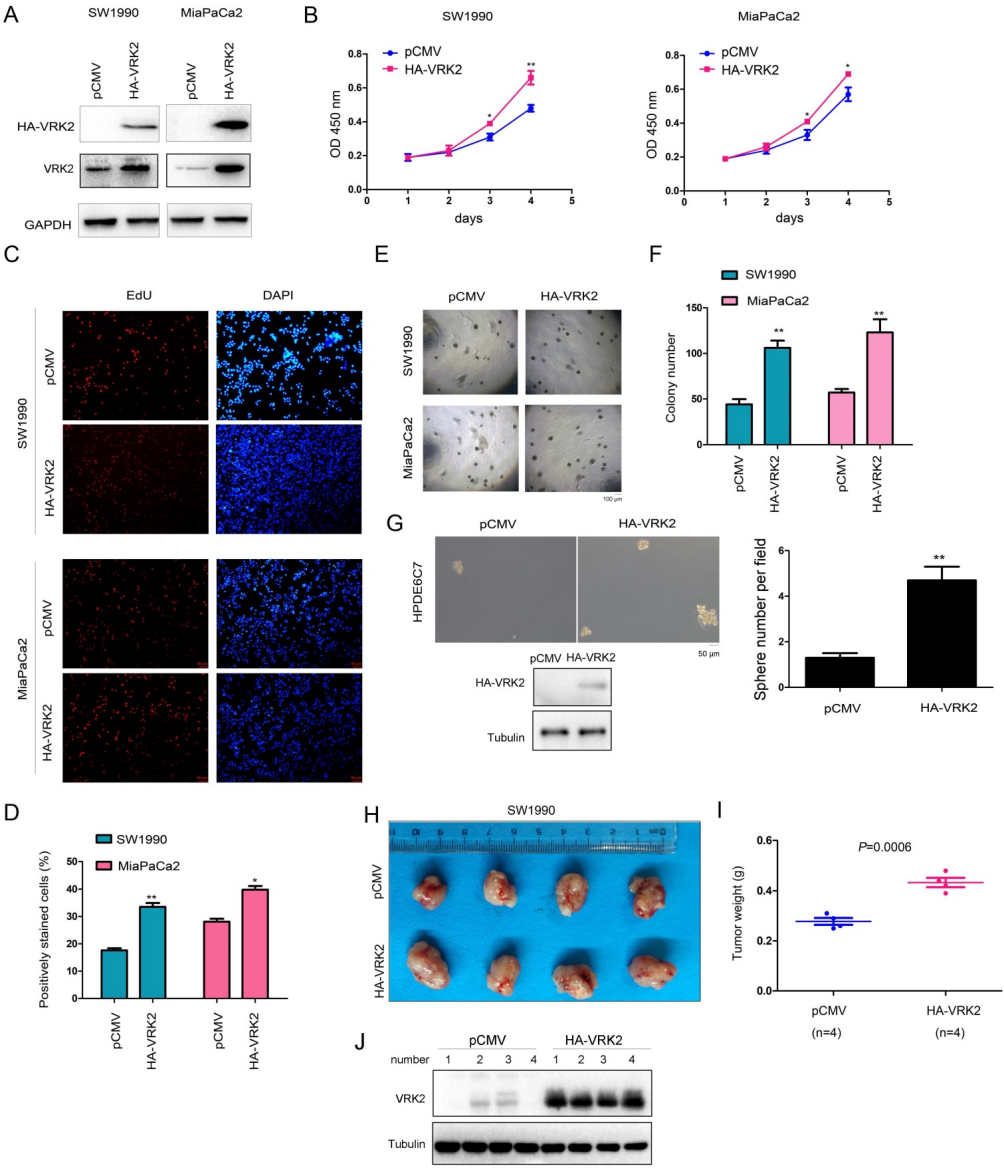
** VRK2 promotes the growth of pancreatic cancer cells.** (**A**) Overexpression of VRK2 in pancreatic cancer cells. Cells were incubated overnight with lentivirus expressing VRK2. Then, cells were selected with puromycin for 3 days, and the resistant cells were pooled for the examination of VRK2 (HA-VRK2) expression. (**B**) A CCK-8 assay was performed to examine the effects of VRK2 overexpression on the growth of pancreatic cancer cells. (**C-D**) An EdU incorporation assay was performed to examine the effects of VRK2 on the proliferation of pancreatic cancer cells. Nuclei were stained with DAPI. EdU-positive cells were counted, and the percentage of EdU-positive cells was calculated. (**E-F**) A soft agar assay was performed to examine the effects of VRK2 on the anchorage-independent growth of pancreatic cancer cells. The colonies were counted, and the data were analyzed. (**G**) A sphere formation assay was performed to examine the effects of VRK2 on sphere formation. (**H-J**) A tumorigenesis assay was performed to examine the effects of VRK2 over-expression on the tumorigenicity of pancreatic cancer cells. Images of the xenografts (**H**), tumor weights (**I**) and the expression of VRK2 (**J**) are shown. *, *P* < 0.05; **, *P* < 0.01.

To reveal the role of VRK2 in the progression of pancreatic cancer, VRK2 (HA-VRK2) was first overexpressed in SW1990 and MiaPaCa2 cells (Fig. 2A). Then, the effect of VRK2 overexpression on the malignant phenotype of pancreatic cancer cells was evaluated. The results of the CCK-8 assay showed that overexpression of VRK2 promoted the growth of SW1990 and MiaPaCa2 cells (Fig. 2B), and the results of the EdU incorporation assay showed that overexpression of VRK2 promoted the proliferation of SW1990 and MiaPaCa2 cells (Fig. 2C-D). Moreover, in the soft agar assay, overexpression of VRK2 promoted the anchorage-independent growth of SW1990 and MiaPaCa2 cells (Fig. 2E-F). Also, in the sphere formation assay, overexpression of VRK2 promoted sphere formation in HPDE6C7 cells (Fig. 2G). In addition, the effect of VRK2 overexpression on the subcutaneous tumorigenic ability of SW1990 cells in nude mice was measured. The results suggested that overexpression of VRK2 in SW1990 cells enhanced their tumorigenic ability (Fig. 2H-J).

**Figure 3:**
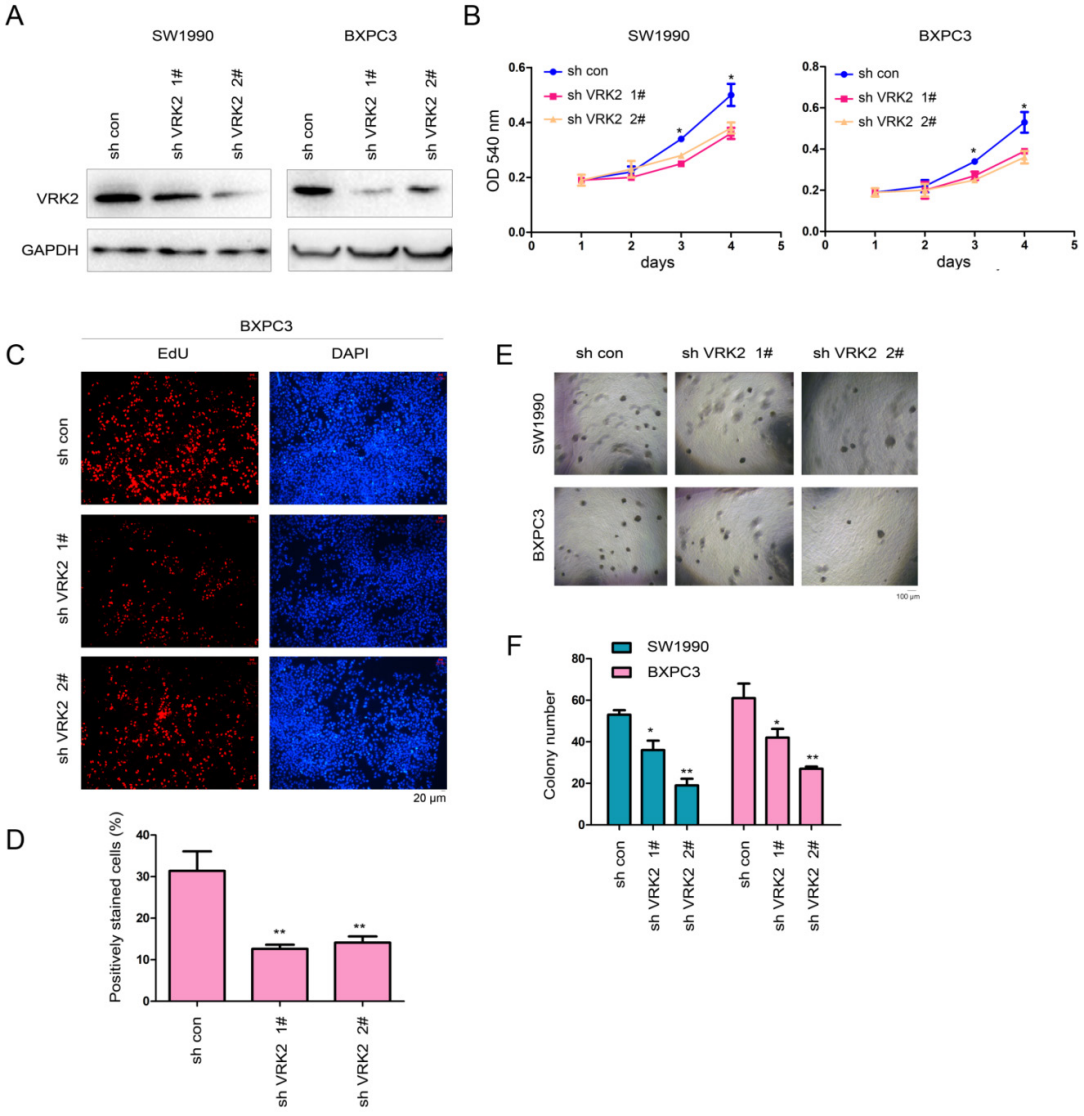
** Knockdown of VRK2 inhibits the growth of pancreatic cancer cells.** (**A**) Knockdown of VRK2 in pancreatic cancer cells. Cells were incubated overnight with lentivirus expressing shRNA targeting VRK2. Then, cells were selected with puromycin for 3 days, and the resistant cells were pooled for the examination of VRK2 expression. (**B**) A CCK-8 assay was performed to examine the effects of VRK2 knockdown on the growth of pancreatic cancer cells. (**C-D**) An EdU incorporation assay was performed to examine the effects of VRK2 knockdown on the proliferation of pancreatic cancer cells. Nuclei were stained with DAPI. EdU-positive cells were counted, and the percentage of EdU-positive cells was calculated. (**E-F**) A soft agar assay was performed to examine the effects of VRK2 knockdown on the anchorage-independent growth of pancreatic cancer cells. The colonies were counted, and the data were analyzed. *, *P*<0.05; **, *P*<0.01.

Then, interference with the expression of VRK2 was achieved in SW1990 and BXPC3 cells using two shRNA sequence (Fig. 3A). The specificity of the two shRNAs was examined (Fig. S1A-B). In the functional assay, the experimental results showed that interfering with the expression of VRK2 inhibited the growth (Fig. 3B), proliferation (Fig. 3C-D), and colony formation of SW1990 and BXPC3 cells (Fig. 3E-F). These observations further demonstrate that VRK2 promotes the progression of pancreatic cancer.

### VRK2 activates the TNFα/NF-kB signaling pathway

**Figure 4:**
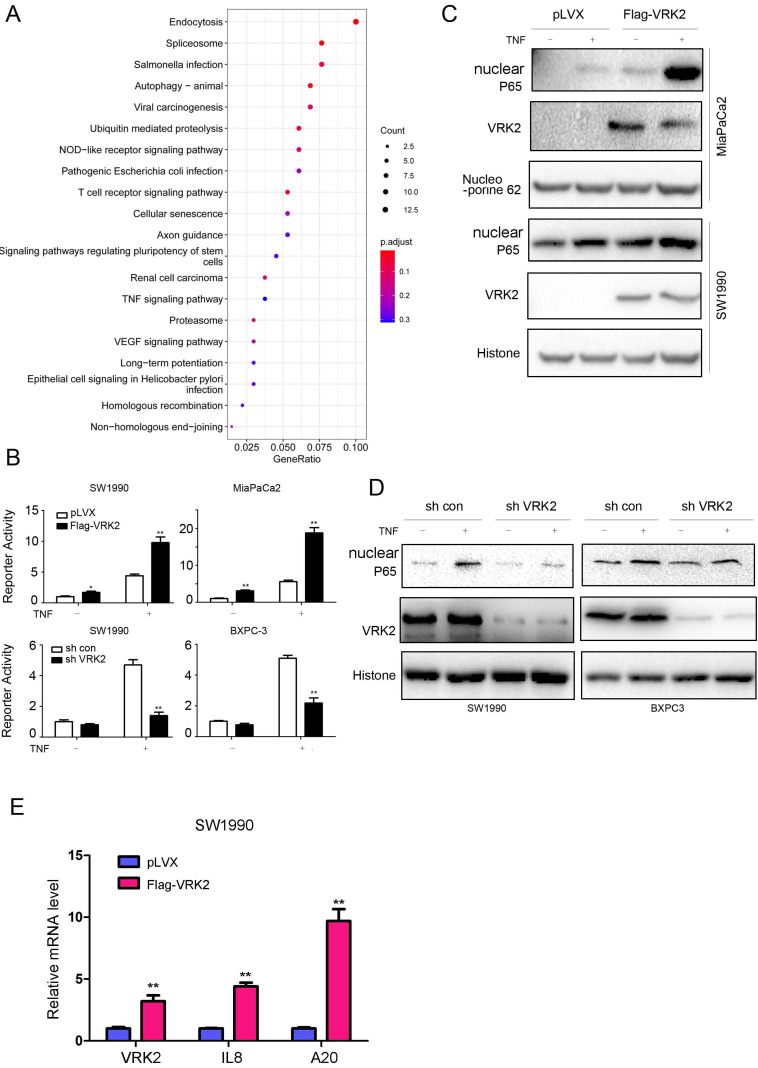
** VRK2 activates the TNFα/NF-κB pathway in pancreatic cancer cells.** (**A**) The enrichment of pathways regulated by VRK2. (**B**) An NF-κB reporter assay was performed to examine the effects of VRK2 overexpression or knockdown on NF-κB signaling activity. (**C-D**) Western blotting was performed to examine the effects of VRK2 overexpression or knockdown on the levels of nuclear P65 protein. Cells were treated with TNFα, and the nuclear protein fraction was isolated. (**E**) qPCR was performed to examine the mRNA levels of target genes downstream of NF-κB signaling. *, *P* < 0.05; **, *P* < 0.01.

To further reveal the molecular mechanism by which VRK2 promotes the progression of pancreatic cancer, KEGG analysis was conducted based on TCGA, a public database, and revealed that VRK2 regulates the TNFα signaling pathway (Fig. 4A). First, a reporter gene was used for verification. As shown in Fig. 4B, VRK2 overexpression cooperated with TNFα in activating the reporter gene, while VRK2 knockdown inhibited the activating effects of TNFα on the reporter gene. Consistent with this finding, VRK2 overexpression promoted nuclear localization of P65 (Fig. 4C), while VRK2 knockdown inhibited nuclear localization of P65 (Fig. 4D). In addition, the expression of VRK2 up-regulated the transcription of A20 and IL8, downstream target genes in the TNFα/NF-kB signaling pathway (Fig. 4E). These research findings indicate that VRK2 activates the TNFα signaling pathway in pancreatic cancer cells.

### VRK2 interacts with IKKβ

**Figure 5:**
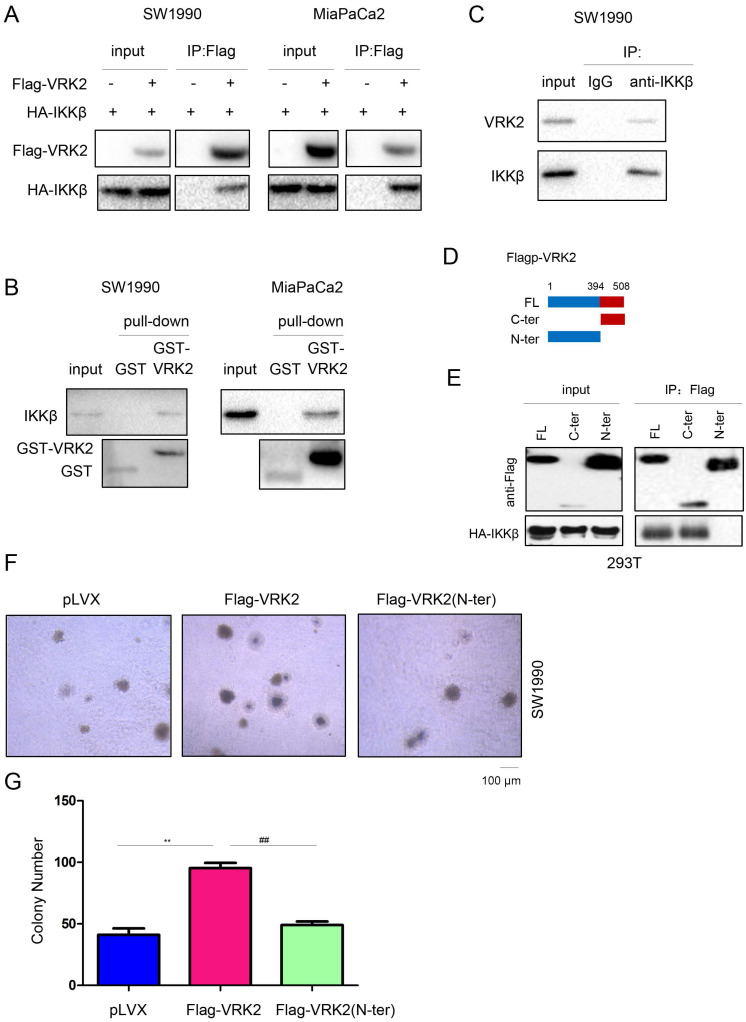
** VRK2 interacts with IKKβ.** (**A**) Immunoprecipitation was performed to examine the interaction between exogenously expressed VRK2 (Flag-VRK2) and IKKβ (HA-IKKβ). Flag-VRK2 and HA-HA-IKKβ expression vectors were transfected into pancreatic cancer cells. Forty-eight hours later, cell lysates were prepared for immunoprecipitation. (**B**) A GST pull-down assay was performed to examine the interaction between the GST-VRK2 fusion protein and endogenously expressed IKKβ. The GST-VRK2 fusion protein was incubated with the lysate of pancreatic cancer cells. Details about the “GST pulldown assay” are described in the “Materials and methods” section. (**C**) Immunoprecipitation was performed to examine the interaction between endogenously expressed VRK2 and IKKβ. (**D-E**) Immunoprecipitation was performed to determine the domain in VRK2 for binding with IKKβ. (**F-G**) A soft agar assay was performed to examine the effects of full-length or truncated VRK2 on the anchorage-independent growth of pancreatic cancer cells. The colonies were counted, and the data were analyzed. **, *P* < 0.01; ##, *P* < 0.01.

To further analyze the molecular mechanism by which VRK2 activates the TNFα/NF-kB signaling pathway, a coimmunoprecipitation (co-IP) assay was conducted to test the interaction between VRK2 and the main components (TNFR, TRAF2, TRAF5, IKKα, IKKβ and IKKγ) of the TNFα/NF-kB signaling pathway. The co-IP results showed that exogenously expressed VRK2 interacted with IKKβ (Fig. 5A), but not TNFR, TRAF2, TRAF5, IKKα or IKKγ (Fig. S2). Furthermore, these results were verified with a GST pull-down assay (Fig. 5B). When co-IP was used to test the interaction between endogenously expressed VRK2 and IKKβ in pancreatic cancer cells, endogenously expressed VRK2 and IKKβ were found to form a complex (Fig. 5C). Moreover, the C-terminal domain of VRK2 was found to be essential for its interaction with IKKβ (Fig. 5D-E). After deletion of the C-terminus, the promotive effect of VRK2 on the colony formation of pancreatic cancer cells was abolished (Fig. 5F-G).

### VRK2 phosphorylates IKKβ

**Figure 6:**
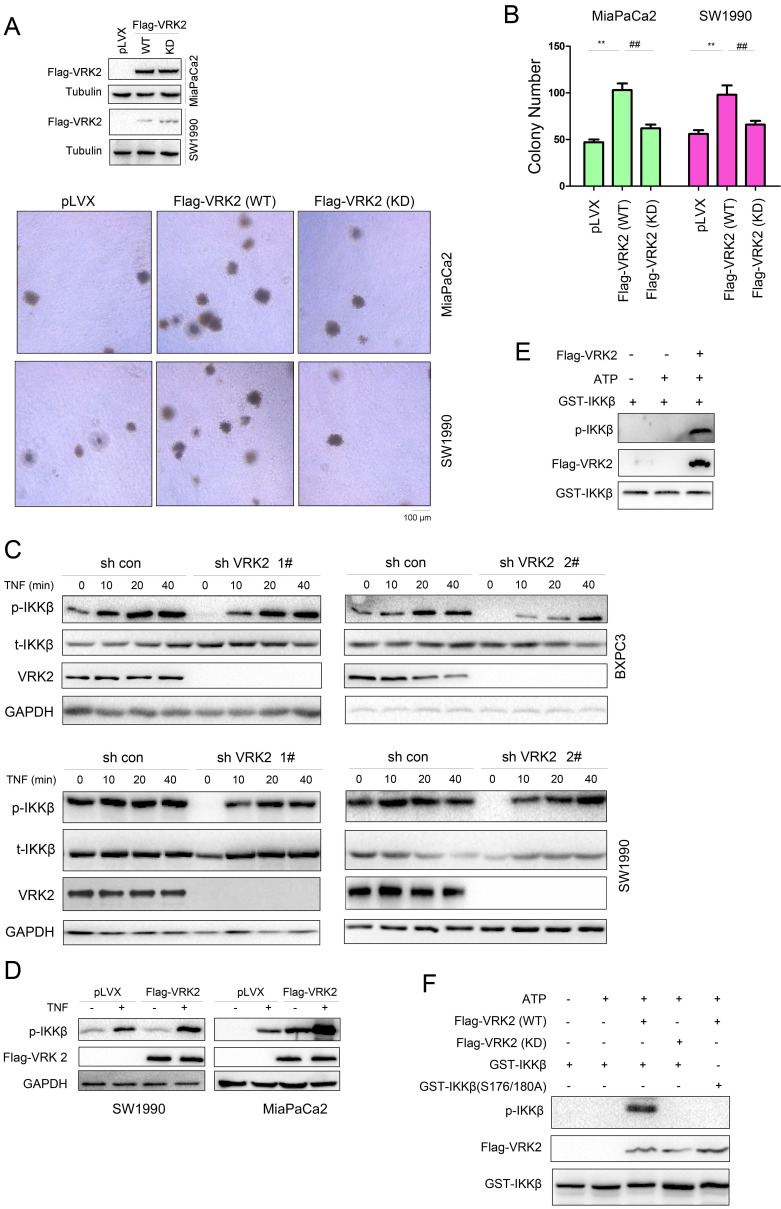
** VRK2 phosphorylates IKKβ.** (**A-B**) A soft agar assay was performed to examine the effects of wild-type and kinase-dead VRK2 (KD) on the anchorage-independent growth of pancreatic cancer cells. The colonies were counted, and the data were analyzed. (**C-D**) After knockdown or overexpression of VRK2 in pancreatic cancer cells, the phosphorylation of IKKβ was examined upon treatment with TNFα. (**E**) An in vitro kinase assay was performed to examine the phosphorylation of the GST-IKKβ fusion protein by VRK2. Details about the in vitro kinase assay are described in the “Materials and methods” section. (**F**) An in vitro kinase assay was performed to examine the phosphorylation of the GST-IKKβ fusion protein by wild-type and kinase-dead VRK2. **, *P* < 0.01; ##, *P* < 0.01.

To study the molecular mechanism by which VRK2 regulates IKKβ, whether the kinase activity of VRK2 is essential for its function in pancreatic carcinoma was first investigated. The kinase-dead mutant of VRK2 (K61A/K169E) failed to promote the colony formation of pancreatic cancer cells as wild-type VRK2 did. This finding indicates that the role of VRK2 in pancreatic cancer depends on its kinase activity (Fig. 6A-B). Interfering with the expression of VRK2 inhibited TNFα-induced phosphorylation of IKKβ (Fig. 6C). VRK2 overexpression cooperates with TNFα to induce the phosphorylation of IKKβ (Fig. 6D). In the in vitro kinase assay, it was observed that VRK2 phosphorylated Ser177/181 of IKKβ (Fig. 6E). Kinase-dead mutants of VRK2 could not phosphorylate Ser177/181 of IKKβ (Fig. 6F).

### VRK2 promotes the malignant phenotype of pancreatic cancer cells by activating IKKβ

**Figure 7:**
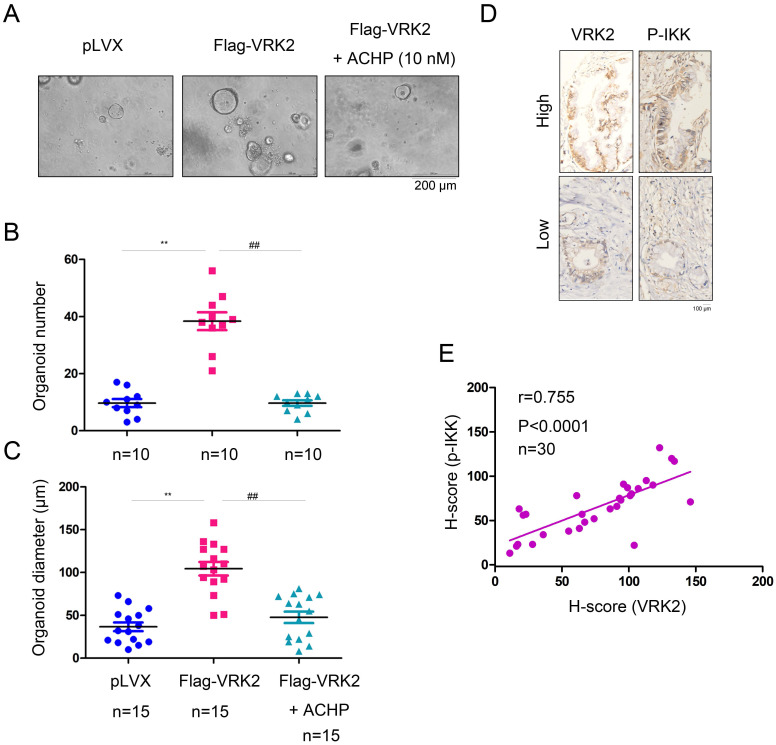
** VRK2 promotes pancreatic cancer by activating IKKβ.** (**A**) The IKKβ inhibitor impaired the growth of organoids induced by VRK2 overexpression. (**B-C**) The number and diameter of the organoid. (**D-E**) The expression of VRK2 and phosphorylation level of the IKKβ protein were examined by IHC, and the correlations were analyzed. **, *P* < 0.01; ##, *P* < 0.01.

To verify whether VRK2 promotes the progression of pancreatic cancer by activating IKKβ, an organoid model of pancreatic carcinoma was established using the ductal tissues derived from the KC mice (Fig. 7A). After overexpression of VRK2 in KC mouse-derived organoids, both the number and diameter of the organoids were increased significantly (Fig. 7B-C). However, after the organoids were treated with ACHP (10 nmol), an IKKβ inhibitor, both the number and diameter of the organoids was decreased (Fig. 7B-C). This result shows that VRK2 promotes the progression of pancreatic cancer through IKKβ. In addition, in the pancreatic cancer sample, the expression of VRK2 was positively correlated with the phosphorylation level of IKKβ (Fig. 7D-E), further indicating the regulatory effect of VRK2 on IKKβ.

## Discussion

**Figure 8:**
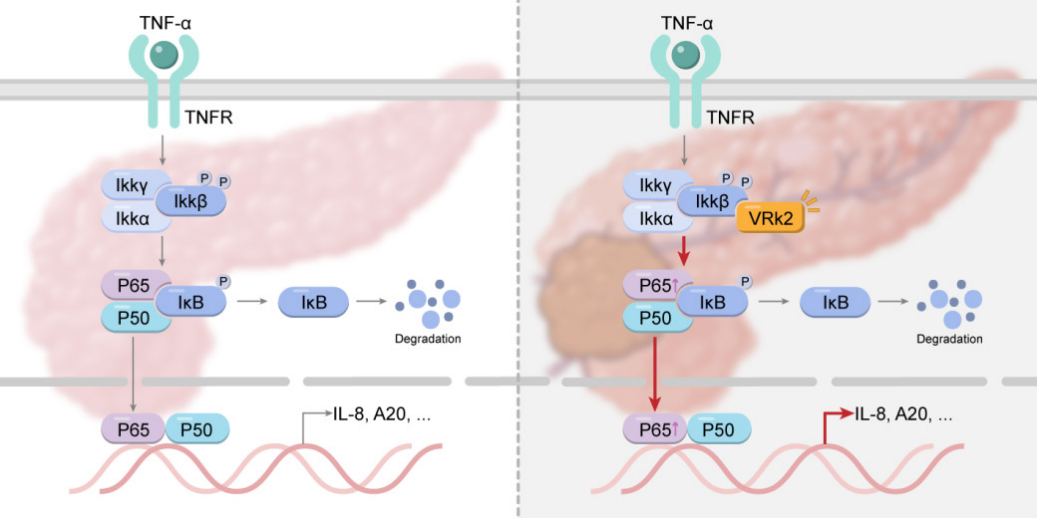
Graphical abstract: VRK2 activates the NF-κB signaling pathway by phosphorylating IKKβ, thereby promoting the progression of pancreatic cancer.

It has been reported that the expression of NF-κB is upregulated in pancreatic cancer cell lines and clinical tissues; moreover, its expression level is significantly correlated with poor prognosis in patients with pancreatic cancer 6, 37. These observations indicate that it is of great importance for the treatment of pancreatic cancer to further study the regulatory mechanism of the NF-κB signaling pathway. The findings of this study suggest that VRK2 activates the transcription of NF-κB by phosphorylating IKKβ, thereby promoting the growth of pancreatic cancer cells and enhancing their tumorigenic ability in vivo (Fig. 8). This study not only reveals the function and mechanism of VRK2 in pancreatic cancer but also provides a potential therapeutic target for pancreatic cancer.

One of the most important findings of this study is the discovery of the direct phosphorylation of IKKβ by VRK2. Some previous studies have shown that Kras^G12D^-driven pancreatic cancer can be inhibited if IKKβ kinase activity is inhibited or the expression of IKKβ is knocked out 38. This also shows that targeting IKKβ is a feasible strategy for the treatment of pancreatic cancer. Since the discovery of IKKβ, continuing research has been carried out on the development of IKKβ inhibitors. The existing IKKβ inhibitors (e.g., ML-120B and TPCA1) cannot be used in clinical practice because of their nonnegligible side effects. According to this study, it seems feasible to develop a VRK2 inhibitor to inhibit the activity of IKKβ upstream.

Another interesting finding of this study is that VRK2 promotes the formation of spheres and organoids. It has been reported that sphere formation is an important means to enrich tumor stem cells 39, 40. Moreover, the formation of organoids also depends on tumor stem cells 41, 42. Therefore, these observations suggest that VRK2 is likely to affect the function of pancreatic cancer stem cells. Pancreatic cancer stem cells play an important role in the occurrence, metastasis, and drug resistance of pancreatic cancer 43. Therefore, it is of great importance to target VRK2 in order to inhibit the progression of pancreatic cancer.

A previous study by Zhu et al. also demonstrated the oncogenic activity of VRK2 in pancreatic cancer 44. However, Zhu et al. did not examine the correlation between VRK2 expression and clinical features. Our study demonstrated positive correlations between VRK2 expression and tumor size, further suggesting the promoting effects of VRK2 in the progression of pancreatic cancer. Moreover, both studies showed that the oncogenic activities of VRK2 were dependent on its kinase activity, although the substrates in these two studies were different. Based on the regulation of PLK1 and IKKβ reported in these two studies, VRK2 might be an important hub of signal transduction in pancreatic cancer. Therefore, VRK2 inhibitors might have therapeutic effects on pancreatic cancer by targeting multiple signaling pathways simultaneously.

Limitations of this study included the following. First, in this study, the functions of VRK2 in pancreatic cancer were examined using a cell model, and knocking out VRK2 in a pancreatic cancer mouse model would provide more insights into its roles. Second, although we have provided evidence that VRK2 activates TNFα/NF-kB signaling in pancreatic cancer cells, when or under what conditions VRK2 controls NF-kB signaling regulation remains largely unknown.

It has been reported that wild-type p53 down-regulates the expression of VRK2 45. p53 is mutated in about 70% PDAC^46^, which provides a good explanation for the up-regulation of VRK2 in the pancreatic cancer. Moreover, hypoxia is been reported as a stress condition that induces VRK2 kinase 47. Considering hypoxia is one of the hallmarks of cancer 48, it is not strange that VRK2 is overexpressed in the pancreatic cancer. On the other hand, hypoxia is reported to activate NF-kB signaling and increases the resistance of human pancreatic cancer cells to apoptosis induced by gemcitabine 49. Therefore, it would be possible that hypoxia activates NF-kB signaling via VRK2.

TNFα can trigger both pro-survival and pro-apoptosis signaling through the NF-kB pathway and the caspase-8 pathway. The cell fate determination mainly depends on the balance between these two pathways. We found that overexpression of VRK2 in pancreatic cancer cells could activate TNFα/NF-kB pathway, leading to a shift from apoptosis to survival in the circumstance of TNFα. This may explain how pancreatic cancer cells tolerate the high level of such potential apoptotic factors. Therefore, further study is needed to explore how VRK2 is incorporated into TNFα/NF-kB signaling.

In conclusion, the findings of this study suggest that the expression of VRK2 is upregulated in pancreatic cancer and that VRK2 activates the NF-κB signaling pathway by phosphorylating IKKβ, thereby promoting the progression of pancreatic cancer. This study indicates that VRK2 is likely to be a therapeutic target for pancreatic cancer.

## Materials and methods

### Cell culture and transfection

Pancreatic cancer cell lines (SW1990, BXPC3, MiaPaCa2, HPAC and CFPAC), normal ductal epithelial cell line (HPDE6C7), and 293T cells were obtained from the Cell Bank of the Chinese Academy of Sciences. Cells were cultured in DMEM. Fetal bovine serum (FBS, 10%, Gibco) and antibiotics (100 U/mL penicillin and 100 µg/ml streptomycin, Gibco) were added to all media. All cells were cultured in a constant temperature incubator (5% CO_2_, 37°C). Cell transfection was performed using Lipofectamine 8000 according to the instruction's manual.

### Overexpression or knockdown of VRK2 expression

To generate the VRK2 expression vector, the open reading frame of human VRK2 cDNA was cloned into the expression vector pCMV-HA and fused to an HA tag (HA-VRK2). For the establishment of stable cell lines, HA-VRK2 plasmids were transfected into the cells using Lipofectamine 8000, and the cells were selected with G418 (400 µg/ml) for two weeks. Alternatively, the open reading frame of human VRK2 cDNA was cloned into the lentiviral vector pLVX and fused to a Flag tag. For the establishment of stable cell lines, cells were infected with the virus and selected with puromycin (2 µg/ml) for three days. The resistant cells were pooled and the expression of exogenous VRK2 was examined.

For VRK2 knockdown experiments, the lentiviral vector pLKO.1 was used to produce small, double-stranded RNA (siRNA) to inhibit the expression of VRK2. The pLKO.1 vector with sh VRK2 or sh con was transfected into 293T cells, and the virus was harvested from the culture medium. The harvested virus was purified by centrifugation at 25,000 g (4 °C, 150 min), and appropriate amounts of virus were used to infect pancreatic cancer cells. After 3 days of infection, cells were selected with puromycin (2 µg/ml, for pLVX vector) for three days. The resistant cells were pooled and the expression of VRK2 was verified. The shRNA sequences were as follows: sh VRK2 1#, 5'- aattaggtatccgaatgttgg-3'; sh VRK2 2#, 5'-aaggctgcaacaaagcaagtc-3'. In order to construct shRNA-resistant VRK2 (VRK2^R^), the nucleotide sequence tta ggt atccgaatgttgg in VRK2 CDS targeted by shVRK2 1# was synonymously mutated into atacgtatgctcg; the nucleotide sequence ggctgcaacaaagcaagtc in VRK2 CDS targeted by shVRK2 2# was synonymously mutated into ggatgtaataaggctagcc.

### Immunohistochemistry

The tissue array was obtained from Shanghai OUTDO Biotech Co., Ltd. After dewaxing and rehydration, tissue sections were put in EDTA solution, and antigen recovery was performed at 100°C for 30 min. After natural cooling to room temperature, an endogenous peroxidase blocker was used to block endogenous peroxidase activity for 15 min. After washing twice in PBS, tissue sections were incubated with the anti-VRK2 antibody (Sigma, HPA047503, 1:100) at 4°C overnight. The next day, the tissue sections were washed twice in PBS and were then incubated with a secondary antibody at room temperature for 1 h. The immunohistochemical reactions were visualized with 3,3,0-diaminobenzidine (DAB), and hematoxylin was used for nuclear staining in all tissue sections.

Both the staining intensity and protein expression level were automatically scored with the inForm 2.4.0 software (PerkinElmer). Ten percent of these images were used to create algorithms with inForm software 2.4.0 (PerkinElmer) to segment tissue area and nuclei. Hematoxylin staining was recognized by the software as the nucleus.

The protein levels of VRK2 were evaluated based on the percentage of positive cells and staining intensity (0, negative; 1+, weak; 2+, moderate; 3+, strong) using the H score. The H score is a product of the percentage of cells in each intensity category (0, 1+, 2+ and 3+). H-score was calculated by the software using the following formula: H-score=3* (% of 3+ cells) + 2* (% of 2+ cells) + 1*(% of 1+ cells).

### EdU incorporation assay

Cells were plated into a 96-well plate, with 20 000 cells in each well. Cell proliferation was detected using a Cell-Light EdU Apollo567 In Vitro Kit (RiboBio, C10310-1). A fluorescence microscope was used to acquire images for analysis. The percentage of positively stained cells was calculated.

### Soft agar assay

At 60-70% confluence, cells were digested, and a cell suspension was prepared. The bottom gel layer (20% FBS, 40% 2× RPMI1640 (Basal Medium Eagle), 40% 1.25% agar) was prepared. Then, 400 μL of the gel was added to each well in a 24-well plate. The 24-well plate with the gel was placed in an incubator at 37°C. The gel was solidified for later use. The top gel layer (25% FBS, 37.5% 2× RPMI1640, 37.5% 1% agar, 0.8% 2 mM L-glutamine) was prepared and mixed evenly with the cell suspension. Then, 400 μl (containing 1000 cells) was added to each well and placed in a constant temperature incubator (37°C, 5% CO_2_) for two weeks. Five fields were selected randomly under a microscope for colony counting.

### Sphere formation assay

Cells were plated into a low-attachment 24-well plate (Corning, 3473#) and cultured with DMEM/F12 containing 20 ng/mL EGF (Sigma, E9644), 20 ng/mL FGF (R&D Systems, 233-FB-025) and 2% B27 (Thermo Fisher Scientific, 17504044) to test sphere formation. Seven days later, images were acquired under a microscope, and the formed spheres were counted.

### Western blot analysis

Cells were washed twice with PBS and lysed on ice with RIPA lysis buffer containing a protease inhibitor and phosphatase inhibitor. The supernatant was collected after the cell lysate was centrifuged, and the protein concentration was quantified using a BCA protein detection kit. Equal amounts of protein were subjected to SDS-PAGE. After separation, the proteins were transferred onto a PVDF membrane and incubated with a specific primary antibody at 4°C overnight. Then, the membrane was incubated with an HRP-conjugated secondary antibody for 1 h. Immunoreactions were detected with a chemiluminescence reagent (Milliwell, WBKLS0050) and analyzed with Image Lab software. The primary antibodies used in this experiment were as follows: anti-VRK2 (Proteintech, 12946-1-AP, 1:1 000), anti-tubulin (Santa Cruz Biotechnology, sc-5286, 1:4 000), anti-Histone H3 (Cell Signaling Technology, 97733s, 1:1 000), anti-Flag (Sigma, F9291; 1:3 000), anti-HA (Sigma, H3663, 1:2 000), anti-P65 (Cell Signaling Technology, 8242s, 1:1 000), anti-IKKβ (Cell Signaling Technology, 2678s, 1:1 000), anti-Phospho-IKKα/β (Ser176/180) (Cell Signaling Technology, 2697s, 1:1 000), and anti-GST (Cell Signaling Technology, 2624s, 1:5 000).

### Reporter assay

Cells were plated into a 12-well plate at a density of 50%. Then, the cells were co-transfected with 0.1 μg of the expression vector, 0.05 μg of the reporter plasmid and 0.02 μg of the Renilla luciferase plasmid. 24 h after transfection, the cells were incubated with TNF for 8 h. Then, a dual-luciferase reporter assay system (Beyotime, RG088M) was used to test luciferase activity. The experiment was repeated three times.

### Nuclear protein extraction

After being washed with precooled PBS, cells were harvested by scraping with PBS. Then, the cells were centrifuged (4°C, 500 × g) for 5 min to remove the supernatant. Buffer A (10 mmol/L HEPES/KOH (pH 7.9), 1 mmol/L DTT, 0.1 mmol/L EGTA, 0.1 mmol/L EDTA, 10% NP-40, 0.2 mmol/L phenylmethylsulfonyl fluoride, protease inhibitor and phosphatase inhibitor) was used for cell resuspension. The suspended cells were lysed on ice for 30 min and were then centrifuged (4°C, 1 000 × g) for 10 min, and the supernatant was taken as the cytoplasmic protein fraction. Buffer B (20 mmol/L HEPES/KOH (pH 7.9), 0.4 mmol/L NaCl, 1 mmol/L DTT and 0.2 mmol/L phenylmethylsulfonyl fluoride, protease inhibitor and phosphatase inhibitor were added to the precipitate for resuspension. The precipitate was lysed on ice for more than 4 hours and was then centrifuged (4°C, 12000 × g) for 15 min, after which the supernatant was taken as the nuclear protein fraction. After quantification of the concentration, the protein was immediately used for western blot analysis.

### Immunoprecipitation

To detect the interaction between exogenous VRK2 (Flag-VRK2) and IKKβ (HA-IKKβ), Flag-VRK2 and HA-IKKβ plasmids were transfected into 293T cells. 48 h after transfection, the cells were lysed with IP lysis buffer (50 mM Tris-HCl (pH 8.0), 150 mM NaCl, 1% NP-40, protease inhibitor and phosphatase inhibitor), and the supernatant was collected. Beads coupled to an anti-Flag antibody (Sigma, A2220) were added to the supernatant for incubation overnight at 4°C. The next day, the beads were washed 3 times in wash buffer (50 mM Tris-HCl (pH 8.0), 150 mM NaCl, and 1% NP-40), 1× loading buffer was added, and the beads were heated at 100°C for 5 min. Then, the supernatant was collected for western blot analysis.

To detect whether there was any interaction between endogenously expressed VRK2 and IKKβ in pancreatic cancer cells, IP lysis buffer containing protease and phosphatase inhibitors was used to lyse cells. Equal amounts of protein were aliquoted, and 0.25 μg of an anti-IKKβ antibody was added for incubation overnight at 4°C. The next day, 40 μL of Protein A/G beads (Bimake, B23202) was added for another incubation overnight at 4°C. The beads were washed 3 times with wash buffer, and 1× loading buffer was then added for western blot analysis.

### GST pulldown assay

IP lysis buffer containing protease and phosphatase inhibitors was used for lysis of pancreatic cancer cells. After centrifugation, the supernatant was collected, and 10 μg of the GST-VRK2 fusion protein was added for incubation overnight at 4°C. The next day, 40 μL of glutathione-Sepharose (Thermo, G2879) was added for another 4-hour incubation at 4°C. The beads were washed 3 times with wash buffer, and 1× loading buffer was then added for western blot analysis.

### In vitro kinase assay

To generate the GST-IKKβ and GST-IKKβ (S176/180A) expression vectors, the open reading frame of human wild-type IKKβ or mutant IKKβ (S176/180A) cDNA was cloned into the expression vector pGEX-4T-1 and fused to a GST tag. BL-21 bacterial cells were transformed with GST-IKKβ and GST-IKKβ (S176/180A) expression vectors.

The Flag-VRK2 plasmid was transfected into 293T cells. Forty-eight hours later, Flag-VRK2 was purified by coimmunoprecipitation (Co-IP) and competitively eluted with a Flag polypeptide to obtain a Flag-VRK2 protein solution. IPTG was used to induce the expression of the GST-IKKβ fusion protein in BL-21 bacterial cells. The bacterial cells were collected, resuspended, and lysed with IP lysis buffer. Then, the lysate was sonicated at 20% power for 9 s and incubated in an ice bath for 1 min. This operation was repeated 3 times. Then, the lysate was centrifuged (4°C, 14 000 rpm) for 10 min, and the supernatant was collected. GST beads (20 μL; GE Healthcare, 17-0756-1) was added to the supernatant for 1-2 h of incubation at 4°C. The beads were washed 3 times in wash buffer. ATP (10 mM; Cell Signaling Technology, 9804S) and 10× kinase buffer (Cell Signaling Technology, 9802S) were added and allowed to react at 32°C for 45 min. Loading buffer was added to stop the reaction and western blot analysis was then performed.

### Organoid formation from single cells

Detailed procedures for isolating normal pancreatic ducts have been described previously 50. In brief, normal and preneoplastic pancreatic ducts were manually picked after enzymatic digestion of the pancreas with 0.012% (w/v) collagenase XI (Sigma) and 0.012% (w/v) dispase (GIBCO) in DMEM containing 1% FBS (GIBCO) and were then seeded into growth factor-reduced (GFR) Matrigel (BD).

For organoid formation from a single cell, organoids were washed with ice-cold cell recovery solution (Corning) and digested into single cells. The cells were gently diluted to approximately 5000 cells/ml using a 1:1 mixture of ice-cold feeding medium and Matrigel. The mixture was added to a 48-well plate at 100 µl per well. In the organoid formation assay, 500 µl of culture medium was added to the chambers of the 48-well plate. The formation and status of the organoids were examined.

### Tumorigenesis assay

Nude mice aged 4-6 weeks were used, with 4 mice in each group. 1×10^6^ control cells and SW1990 cells with overexpression of VRK2 were injected subcutaneously at each point. The mice were killed in the sixth week after the start of the experiment to harvest tumors. The study was approved by the Animal Ethics Committee of the Zhejiang University.

### Statistical analysis

Data was expressed as mean ± SD. The data were analyzed using the* t* test. The Chi-squared test was conducted to analyze the relationship between the clinicopathological data and the VRK2 scores. A survival curve was plotted by the Kaplan-Meier method, while the log-rank test was used for analysis. GraphPad Prism 8 and SPSS 17.0 were used for statistical analysis.

## Supplementary Material

Supplementary figures.

## Figures and Tables

**Table 1:** The correlation between the VRK2 expression and clinical features.

Characteristic	Total	VRK2 Expression		χ2	p-Value
		Low (n=45)	High (n=36)		
Gender					
Male	51	27	24	0.381	0.645
Female	30	18	12		
Age					
≤60	41	23	18	2.374	0.305
>60	40	22	18		
CA19-9 (U/ml)					
≥37	63	37	26	1.157	0.298
37<	18	8	10		
CEA (ng/ml)					
≥5	27	19	8	3.600	0.064
<5	54	26	28		
Tumor Size (cm^3^)					
>3	14	12	2	6.235	0.017*
≤3	67	33	34		
Alcohol					
Yes	18	7	11	2.604	0.118
No	63	38	25		
Smoking					
Yes	21	7	14	5.670	0.023*
No	60	38	22		
TNM Stage					
ⅠB, ⅡA-ⅡB	16	9	7	0.004	1.000
ⅢA-ⅢB,Ⅳ	65	36	29		
Location					
Head	50	29	21	0.316	0.648
Tail	31	16	15		
Lymph node Metastatic					
Yes	34	16	18	1.713	0.258
NO	46	28	18		
P53 Expression					
High	34	19	15	0.003	1.000
Low	47	26	21		
Ki67 Expression					
High	62	31	31	3.304	0.112
Low	19	14	5		

*, *P* < 0.05.
