# Supplementary material for: VRK2 activates TNFα/NF-κB signaling by phosphorylating IKKβ in pancreatic cancer
Source: Int J Biol Sci. 2022 Jan 9;18(3):1288–302. doi: 10.7150/ijbs.66313 (PMC8771851; doi:10.7150/ijbs.66313)
Supplement: Supplementary file 1 — Supplementary figures. [file ijbsv18p1288s1.pdf]

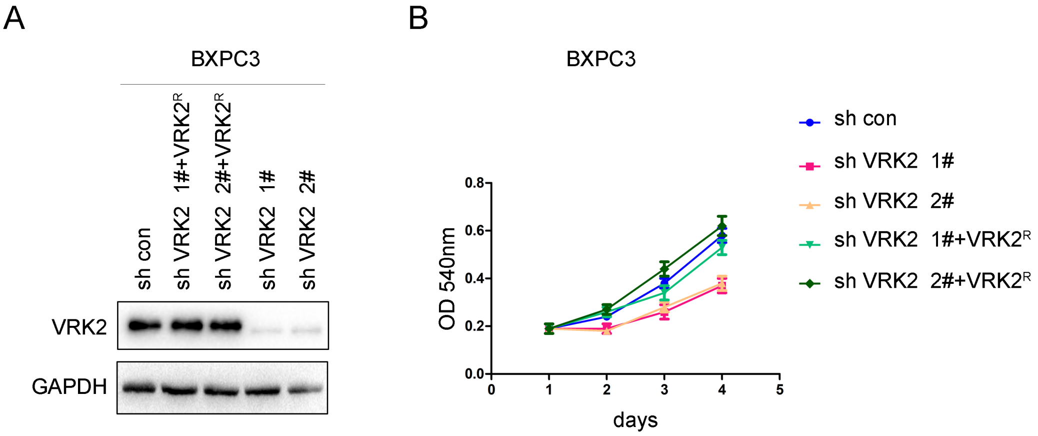

Fig. S1. The specificity of shRNAs for the knocking out VRK2 was examined.
(A) Western blot was performed to examine the levels of VRK2 protein in BXPC3 cells.
(B) CCK8 assay was performed to examine the growth of BXPC3 cells.

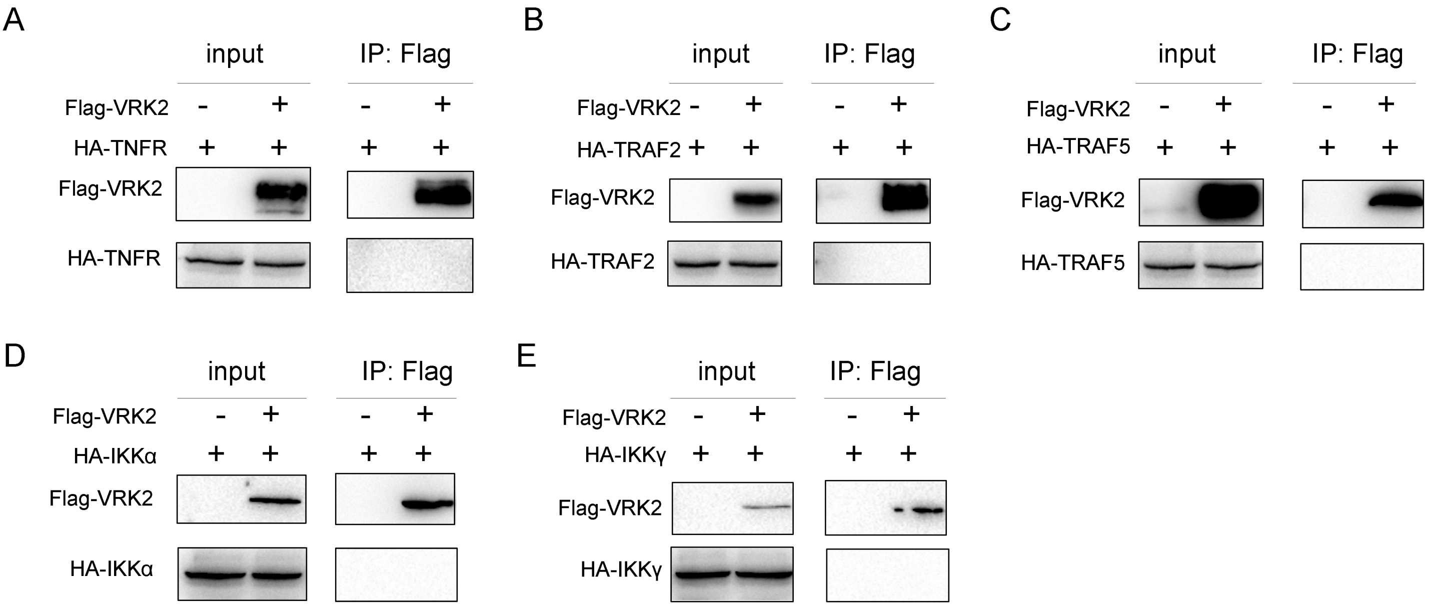

Fig. S2. The interaction between VRK2 and the major components of TNFα/NF-kB pathway was examined by Co-IP. The indicated expression vectors were transfected into 293T cells.
48 hours after transfection, the Co-IP was performed to examine the interaction between Flag-VRK2 and TNFR (A), TRAF2 (B), TRAF5 (C), IKKα (D), IKKγ (E).
